# Substance use and misuse among children and youth with mental illness: A pilot study

**DOI:** 10.1007/s40211-017-0231-4

**Published:** 2017-06-21

**Authors:** V. Herz, N. Franzin, J. Huemer, D. Mairhofer, J. Philipp, K. Skala

**Affiliations:** Department of Child and Adolescent Psychiatry, Medical University of Vienna, Spitalgasse 23, 1090 Vienna, Austria

**Keywords:** Adolescents, Mental illness, Alcohol, Nicotine, Illicit substances, Jugendliche, Psychische Erkrankung, Alkohol, Nikotin, Illegale Drogen

## Abstract

**Objective:**

The aim of this study was to examine prevalence, patterns and predictors of substance use among a sample of adolescent psychiatric inpatients.

**Methods:**

Participants included 25 minors aged 12–17 years admitted to an Austrian department of child and adolescent psychiatry. Lifetime use, initiation, frequency and quantity of substance use, sociodemographic, family and school-related data were collected by self-report measures. Substance use disorders were detected using CAGE (a screening instrument for problem drinking) and FTND (Fagerström Test For Nicotine Dependence). Clinical characteristics were extracted from medical records.

**Results:**

Lifetime prevalence of any substance use (76%) and regular use (32%) were common. Prevalence was high for alcohol (76%), nicotine (44%) and illicit drug use (36%). Older age was associated with tobacco (*p* = 0.023), drug (*p* = 0.021) and cannabis use (*p* = 0.015) and regular use of psychotropic substances (*p* = 0.027). Family dysfunction predicted regular (*p* = 0.035) and cannabis use (*p* = 0.02). History of trauma prognosticated regular (*p* = 0.047) and tobacco use (*p* = 0.011). Use of any substance (*p* < 0.001) as well as regular use (*p* = 0.026) were significantly associated with peer substance use. Consuming adolescents were more likely to show academic failure, school absenteeism and behavioral problems. Alcohol (*p* = 0.02), drug (*p* = 0.017) and regular substance use (*p* = 0.007) were linked to suicidal ideation. A remarkable relationship between affective as well as externalizing disorders and alcohol, nicotine and drug use was found.

**Conclusions:**

Substance use is highly prevalent among youth with mental illnesses and associated with psychosocial consequences. These data highlight the need to carefully explore this population at high risk.

## Zusammenfassung

**Ziel:**

Ziel dieser Studie war die Analyse der Prävalenzen des Konsums psychotroper Substanzen, der Konsummuster sowie der Prädiktoren des Konsums innerhalb eines Kollektivs psychisch kranker Adoleszenter in stationärer psychiatrischer Betreuung.

**Methodik:**

Die Population bestand aus 25 psychisch kranken Adoleszenten (12–17 Jahre), die an der Universitätsklinik für Kinder- und Jugendpsychiatrie des AKH Wien stationär aufgenommen waren. Mittels Selbstbeurteilungsbögen wurden Lebenszeitprävalenzen, Einstiegsalter, Häufigkeit und Menge des Substanzkonsums sowie soziodemographische, familiäre und schulbezogene Daten ermittelt. Symptome eines Substanzmissbrauchs (SUD) wurden mittels CAGE (ein Kurzfragebogen für problematischen Alkoholkonsum) und FTND (Fagerström Test For Nicotine Dependence) erfasst. Ferner wurden klinische Daten aus medizinischen Unterlagen entnommen.

**Ergebnisse:**

Insgesamt 76 % des Kollektivs konsumierten eine psychotrope Substanz, 32 % konsumierten regelmäßig. Es ergaben sich hohe Prävalenzen für Alkohol- (76 %), Nikotin- (44 %) und Drogenkonsum (36 %). Ein höheres Alter war assoziiert mit Nikotin- (*p* = 0,023), Drogen- (*p* = 0,021) und Cannabiskonsum (*p* = 0,015) sowie mit regelmäßigem Konsum einer oder mehrerer psychotroper Substanzen (*p* = 0,027). Dysfunktionale Familienverhältnisse und Traumatisierung waren signifikante Prädiktoren für regelmäßigen Substanzkonsum. Eine konsumierende Peergroup nahm Einfluss auf die Prävalenz des Substanzkonsums (*p* < 0,001) und des regelmäßigen Konsums (*p* = 0,026). Ferner konnten signifikante Beziehungen zwischen Substanzkonsum und Schulversagen, problematischen Verhaltensweisen sowie suizidalen Intentionen aufgedeckt werden. Bemerkenswerte Relationen bestanden zwischen Substanzkonsum und externalisierenden sowie affektiven Störungen.

**Schlussfolgerungen:**

Es konnten hohe Prävalenzen des Konsums psychotroper Substanzen innerhalb des Kollektivs psychisch kranker Adoleszenter und damit verbundene psychosoziale Konsequenzen aufgedeckt werden. Die Ergebnisse unterstreichen die Notwendigkeit einer sorgfältigen Exploration dieser Hochrisikopopulation.

## Introduction

Adolescence represents a developmental period which appears to be essential regarding substance use initiation and the development of mental and behavioral disorders due to psychoactive substance use [1, 2]. There is strong evidence that adolescent substance use can be connected to a number of negative cognitive, psychosocial or mental health consequences [2–5] and that psychiatric disorders are risk factors for the involvement in adolescent substance use [5–9].

In epidemiological studies on alcohol, nicotine and illegal drug use among European youth, prevalence of psychoactive substance use was found to be high [10, 11]. Adolescents aged 15 to 16 reported using alcohol at a rate of 80%, nicotine at 46% and illicit drugs at 18% at least once [10]. Prevalence within adolescents in Austria was found to be even higher [12]. Regarding alcohol, abuse or even dependence could be found among adolescents [2]. Furthermore, heavy episodic drinking (“binge drinking”), described as the consumption of 5 or more drinks within one drinking event, is the dominant type of alcohol misuse in youngsters [2]. Nicotine consumption shows the highest rates in terms of regular use. One in five adolescents in Austria smokes daily [12]. Cannabis was the most frequently used illicit drug [12–14], with prevalence of lifetime use being lower in Austria (16%) than in other European countries (20%) [10, 12]. Use of other illicit drugs was less common among adolescents with prevalence of 1–2% [10, 13]. The mean age of initiating alcohol use was 13 years, [11, 12, 15], that of smoking the first cigarette 14 years [13]. Illicit drug use usually started at an older age, very early consumption was found among 3% of users [10].

In an attempt to explain the development of substance-related behavior, previous studies described several psychosocial risk factors predisposing alcohol, nicotine and illegal drug use among the adolescent population. Firstly, chance of substance use and misuse increased with age [11, 16]. Secondly, physical, sexual and mental abuse as well as dysfunctional families have been identified as being related to substance use in adolescents [17]. Thirdly, peer influence as another strong determinant was described [18]. Further research has demonstrated that adolescent substance use increased the risk of school failure [2] and suicide attempts [2, 8]. Substance using adolescents were more likely to be involved in serious risk behavior, like being involved in criminal activities, fights or drunk driving [1, 2].

Several studies have evaluated a noteworthy relationship between substance use and psychopathology. On the one hand, adolescents suffering from mental and behavioral disorders due to psychoactive substance use were more likely to show symptoms of mental disorders [8]; on the other hand, psychopathology predicted substance use [19]. Elevated rates of alcohol, nicotine and illicit drug use and misuse were found among adolescents entering psychiatric care compared to their counterparts in the general population. Lifetime use rates of alcohol in adolescent psychiatric population had been reported to reach levels between 45% [8], 75% [20] and almost 100% [21]. Adolescents suffering from mental disorders also had significantly higher lifetime use rates of nicotine with values ranging from 60 to 90% [20, 22, 23]. Likewise, mentally ill adolescents were significantly more likely to use illicit drugs [8]. Furthermore, numerous investigations concluded that within samples of mentally ill youth significant differences in substance use rates could be found. Attention deficit/hyperactivity disorder (ADHD), conduct and personality disorder as well as affective disorders have been linked to elevated substance use and misuse [8, 23, 24]. In contrast, individuals suffering from restrictive eating disorders were significantly less likely to report lifetime prevalence of alcohol, nicotine and drug use compared to other diagnostic groups [25, 26].

The aim of the current study was to investigate prevalence, pattern and frequency of alcohol, nicotine and illicit drug use in a clinical population of adolescents suffering from mental disorders in Vienna, Austria. Psychosocial correlates of substance use behavior should be targeted with regard to age, history of trauma, family dysfunction or suicidal ideation as well as peer influence. Another purpose of this study was to examine psychosocial consequences of substance use and to determine differences between different diagnostic groups. Based upon previous research, we expected high prevalence of substance use and misuse as well as risky pattern of use among our sample of adolescent psychiatric inpatients exceeding findings from the adolescent general population. In addition, we assumed to find subpopulations with elevated risk, i. e., subjects with history of trauma, dysfunctional families or suicidal ideation, subject reporting peer substance use, as well as older adolescents and adolescents suffering from externalizing or affective disorders. Finally, we speculated to find significant differences between using and non-using respondents regarding psychosocial consequences like school failure or problem behavior.

## Methods and statistics

We included patients aged between 11 and 19 years admitted to a child and adolescent inpatient psychiatric unit at the Medical University of Vienna. Data collection was performed between May 2015 and April 2016. Exclusion criteria were inadequate language skills, poor cognitive function, short-term admission due to crisis intervention and lack of consent. For patients who had more than one inpatient admission during the 12-month period of data collection only the first consultation was considered.

Lifetime use rates and initiation, frequency and quantity of alcohol, nicotine and illicit drug use were collected by self-report measures. In addition, sociodemographic, family and school-related data, including peer substance use, family dysfunction, academic achievement and substance use related behavioral problems were queried. Alcohol-related behavior was detected using the CAGE questionnaire (Cutting down, Annoyance by criticism, Guilty feeling, Eye-openers), a 4-item scale proved to have good sensitivity and specificity [27]. Nicotine consumption was investigated using Fagerström Test of Nicotine Dependence (FTND), a 10-item questionnaire highly reliable in rating physical dependence to nicotine [23]. Patients’ answers were objectified using alcohol and carbon monoxide breath analyzer tests [28], %CDT (Carbohydrate-Deficient-Transferrin-Asialotransferrin) as a serological marker to determine recent alcohol consumption [29] and urine screening for psychoactive substances [19]. Clinical diagnosis according to ICD10 and several factors known as risk factors including history of trauma (physical/sexual abuse, mobbing, loss of a first degree relative, refugee with traumatic experience) and suicidal ideation were extracted from medical paper files. Written informed consent was obtained by participants and their legal guardians. The study was approved by the Ethics Committee of the Medical University of Vienna (EK 1080/2014).

IBM SPSS Statistics 23.0 software was used for analyzing data; 2 × 2 contingency tables were calculated and, due to a small sample size, Fisher’s exact test was used to examine differences in sociodemographic factors (i. e., history of trauma, family dysfunction, suicidal ideation, peer substance use, school absenteeism, problem behavior) between substance using and non-using youth as well as to examine any effects of different psychiatric disorders. T‑test and Mann–Whitney U test for not normally distributed variables and small sample size were used to compare arithmetic means (mean age, school grade). Regression analyses were performed to investigate the effect of risk factors on substance use including age, family dysfunction, peer substance use or trauma. *P* values of less than 0.05 were considered significant.

## Results

### Demographics and epidemiology

Overall 69 adolescents aged between 12 and 18 years (*M* = 15.1, *SD* = 1.436) were admitted within the 12-month period of observation. Only 25 (36.3%) individuals who all met criteria of at least one psychiatric disorder other than substance use disorders, participated in this study. The sample consisted of 21 females (84%) and 4 males (16%) with a mean age of 14.4 ± 1.39 years (range 12–17 years). Subjects suffering restrictive subtype of AN (Anorexia Nervosa) (ICD10 F50.0) were disproportionally (52%) represented among this sample. Other diagnoses comprised in the sample were mood (40%) and neurotic disorder (36%) followed by externalizing disorders (28%), including behavioral and personality disorder.

Lifetime (76%) and regular use (32%) of any substance as well as the use of more than one legal or illicit drug (48%) were common. Lifetime use of alcohol was reported by 76%, whereas nicotine (44%) and illicit drugs (36%) were tried less frequently. In all, 16% met diagnostic criteria of substance use disorder (SUD) reaching two or more positive answers in CAGE or more than three items in FTND. Concerning the use of alcohol, risky patterns of consumption such as intoxication (40%), heavy episodic drinking (36%) or regular use (20%) were common. The most frequently used illicit drug was cannabis (32%) followed by cocaine (16%), “legal highs” (8%), Methylendioxy-N-methylamphetamin (MDMA) (4%), benzodiazepines, amphetamines and opioids. The mean age of onset was 12.61 years (*SD* = 1.5) for alcohol, 12.85 years (*SD* = 1.292) for nicotine and 14.38 years (*SD* = 1.598) for illicit drug use. Older age was associated with tobacco (*p* = 0.023), drug (*p* = 0.021) and cannabis use (*p* = 0.015) and regular use of psychotropic substances (*p* = 0.027). Most of the subjects made their first experience at the age of 13 or younger. For details see Table 1.

**Table 1:** Prevalence and patterns of use of different substances in whole sample (*N* = 25) and in subjects suffering from different psychiatric disorders

	Subjects *N* = 25	Externalizing disorders *N* = 7	Affective disorders *N* = 10	Anorexia nervosa *N* = 13
*Any substance use*				
Lifetime prevalence	76%	85%	100% (*p* = 0.051)	61.5%
Age of onset	*M* = 12.44, *SD* = 1.54	–	–	–
Regular use	32%	–	–	–
Substance use disorder (CAGE/FTND)	16%	43% (*p* = 0.053)	20%	**0% (φ =** **−0.45/** ***p*** **=** **0.039)**
*Alcohol use*				
Lifetime prevalence	76%	85%	100% (*p* = 0.051)	61.5%
Age of onset	*M* = 12.61, *SD* = 1.5	*M* = 12.4, *SD* = 1.95	*M* = 12.44, SD = 1.59	*M* = 12.75, *SD* = 1.58
30-day prevalence	32%	–	–	–
Alcohol quantity per month	88.7g (range 12–192g)	–	–	–
Regular use ≥ once a week	20%	–	–	–
Lifetime prevalence of intoxication	40%	–	–	–
Binge drinking	36%	–	–	–
CAGE ≥ 2points	12%	–	–	–
*Nicotine consumption*				
Lifetime prevalence	44%	42%	**70% (** ***p*** **=** **0.049)**	**23% (φ =** **−0.44/** ***p*** **=** **0.047)**
Age of onset	*M* = 12.85, *SD* = 1.29	*M* = 13.5, *SD* = 2.1	*M* = 13.33, *SD* = 1.03	*M* = 13.33, *SD* = 0.58
30-day prevalence	28%	–	–	–
Number of cigarettes smoked per month	314 (3–750)	**533,** *** p*** **=** **0.012**	–	–
Daily use	24%	–	–	–
FTND ≥ 4points	16%	–	–	–
Carbon monoxide breath analyzer >6ppm	4%	–	–	–
*Illicit drug use*				
Lifetime	36%	57%,* p* = 0.053	50%	**15% (φ =** **−0.45/** ***p*** **=** **0.041)**
Age of onset	*M* = 14.38, *SD* = 1.598	*M* = 14.33, *SD* = 2.3	*M* = 14.6, *SD* = 1.67	*M* = 15.5, *SD* = 0.71
Urinalyses positive	12%	–	–	–
Lifetime use of cannabis	32%	–	–	–
Regular use of cannabis	12%	–	–	–
Lifetime use of other drugs than cannabis	16%	–	–	–

*SD* standard deviation, *M*Mean, *bold highlighting* for significant results, *g* gram (of alcohol)

### Potential risk factors of substance use

The use of nicotine* (p* = 0.023), illicit psychoactive substances (*p* = 0.021) and cannabis (*p* = 0.015) likewise regular substance use (*p* = 0.027) significantly increased with age. There was a significant relationship between a history of trauma and regular use (*p =* 0.028) as well as the use of more than one substance (*p =* 0.015). Two thirds of traumatized patients showed risky patterns of consumption. History of trauma significantly predicted regular (odds ratio [*OR*] = 11.744, *p* = 0.047) and multiple drug use (*OR* = 11.989, *p* = 0.025) as well as lifetime nicotine consumption (*OR* = 15.510, *p* = 0.011). Odds for regular use increased 15-fold (*OR* = 15.105, *p* = 0.035) and odds of lifetime use of cannabis increased 12-fold (*OR* = 12.081, *p* = 0.02) in mentally ill youth reporting family dysfunction. There was also a significant association between peer consumption and substance use (*p* < 0.001). For details see Table 2.

**Table 2:** Risk factors associated with prevalence of substance use and patterns of high risk use

Risk factor	Negation	Affirmation	Test statistic^a^	*p*-value (Fisher)
*Trauma*				
No substance use	6	0	χ ^2^ = 5.26	0.051
Any substance use	9	10		
No use of more than one substance	11	2	χ ^2^ = 6.84	**0.015**
Use of more than one substance	4	8		
No regular use	13	4	χ ^2^ = 6.01	**0.028**
Regular substance use	2	6		
*Family dysfunction*				
No substance use	5	1	χ ^2^ = 0.50	0.637
Any substance use	13	6		
No use of more than one substance	12	1	χ ^2^ = 5.54	**0.03**
Use of more than one substance	6	6		
No regular use	15	2	χ ^2^ = 6.95	**0.017**
Regular substance use	3	5		
*Peer substance use*				
No substance use/no use of alcohol	6	0	χ ^2^ = 16.78	**<0.001**
Any substance use/alcohol use	2	17		
No regular use	8	9	χ ^2^ = 5.54	**0.026**
Regular substance use	0	8		
No use of more than one substance	8	5	χ ^2^ = 10.86	**0.002**
Use of more than one substance	0	12		
No nicotine use	8	6	χ ^2^ = 9.24	**0.003**
Nicotine use	0	11		
No illicit drug use	8	8	χ ^2^ = 6.62	**0.022**
Illicit drug use	0	9		

^a^Test statistic based on 2 × 2 contingency table

### Psychosocial and mental health consequences

Non-using adolescents had a significantly better school performance than their contemporaries reporting multiple drug consumption (*p* = 0.019) or meeting diagnostic criteria of SUD (*p* = 0.047). Compared with 10% of infrequent users, three quarters of regular substance users reported problem behavior (Fisher’s exact test *p* = 0.004) and school absenteeism was also significantly more frequent in regular substance users (*p* = 0.03). The number of subjects who reported lifetime suicidal ideation was significantly higher in regular users (77%) when compared to the group of infrequent users (25%; *p =* 0.007). For details see Table 3.

**Table 3:** Factors and psychosocial consequences associated with substance use

Psychosocial consequences	Negation	Affirmation	Test statistic^a^	*p*-value (Fisher)
*School absenteeism*				
No regular use	11	6	χ ^2^ = 5.94	0.03
Regular substance use	1	7		
*Substance use-related problem behavior*				
No regular use	15	2	χ ^2^ = 9.99	0.004
Regular substance use	2	6		
No use of more than one substance	12	1	χ ^2^ = 7.35	0.011
Use of more than one substance	5	7		
No substance use disorder	17	4	χ ^2^ = 10.12	0.006
Substance use disorder	0	4		
*Suicidal tendencies*				
No regular use	13	4	χ ^2^ = 9.04	0.007
Regular substance use	1	7		
No alcohol use	6	0	χ ^2^ = 6.20	0.02
Alcohol use	8	11		
No illicit drug use	12	4	χ ^2^ = 6.51	0.017
Illicit drug use	2	7		

^a^Test statistic based on 2 × 2 contingency table

### Differences in prevalence of substance use within diagnostic groups

Although prevalence of alcohol consumption (85%, *p* = 0.637) and use of illicit drugs (57%, *p* = 0.053) were high within subjects diagnosed with externalizing disorders, differences to other diagnostic groups were not significant. Patients of this diagnostic group however had a significantly higher nicotine consumption prior to hospital admission (*p* = 0.012). Affective disorders significantly increased the risk of initiating nicotine use (*p =* 0.049). Individuals suffering from restricting type of AN were significantly less likely to use nicotine (*p* = 0.047) or illicit drugs (*p* = 0.041). None of the anorectic patients met the CAGE and/or FTND cut-off score (*p* = 0.039).

## Discussion

This study investigated substance use in 25 minors admitted to an inpatient psychiatric facility. Three quarters of our sample had reported lifetime consumption of alcohol, which is comparable to findings within German psychiatric inpatient populations [20, 21], but higher than French and Norwegian investigations describing lower prevalence of 45 to 60% [8, 23]. Also in agreement with previous findings, 20% of our sample used alcohol regularly, whereas our sample was more likely to experience alcohol intoxication (40% vs. 25%) [20].

Lifetime nicotine consumption was high with 50%. Most other investigations however found even higher rates of up to 90% in mentally ill adolescents [21–23].

Previous studies have indicated that the use of illicit drugs (50–60%) [20, 21] and especially cannabis use (50%) [9, 21, 30] are common among mentally ill adolescents. In our sample two thirds had already consumed an illicit drug, with cannabis being most frequently used. Regarding prevalence of the use of other illicit drugs than cannabis, our data correspond with the rate of 10% determined by other investigations [23, 30]. Moreover, the study sample showed an earlier age of initiation of consumption of any substance investigated, once again indicating high vulnerability to substance use and elevated risk of SUD in this clinical sample [1, 8].

As previously described [17, 23], subjects having experienced trauma or family dysfunction were significantly more likely to use alcohol, nicotine or drugs or to exhibit risky patterns of consumption. Also in agreement with findings in previous studies [18, 31], we identified peer groups as significant determinants. While each non-user denied peer substance use, adolescents affirming risky patterns of use as well as nicotine or drug consumption all had drug-using peers. High peer influence regarding substance use most likely originates from greater availability of legal and illicit drugs and social pressure.

Concerning psychosocial consequences, subjects reporting multiple consumption or meeting SUD criteria had significantly lower school performance and were more often involved in problem behavior like school absenteeism or legal conflicts [2, 23, 32]. These results are once again in line with previous studies, reporting that mentally ill adolescents consuming in risky pattern were at high risk to develop substance-related problem behavior [2, 23] and had significantly lower school performance [2, 32] than nonconsumers.

A history of suicidal behavior was also significantly linked to regular substance use as well as to alcohol and illicit drug use; all patients reporting a history of suicide ideation were alcohol users. Similar results have been described previously [23, 33].

In investigating the impact of externalizing and affective disorders on consumption patterns, we found that subjects suffering from externalizing disorders showed higher prevalence of alcohol (85%) and drug use (57%) compared to subjects with other diagnosis. Surprisingly, rates of nicotine use did not differ between diagnostic groups which is in contrast with earlier findings [8, 20, 22]. As previously reported [34, 35], we also found a significant association of cigarette smoking and affective disorders. More than two thirds of respondents with affective disorders had smoked at least once, this prevalence rate being twice as high as recently described [8].

While earlier reports indicate that adolescents with AN had a lifetime history of alcohol use of about 20% [25], alcohol use among our sample was stunningly high with 60%. Subjects suffering from a restrictive eating disorder were however significantly less likely to have tried nicotine or illicit drugs compared to other diagnostic groups. Consistent with previous findings [26], this diagnostic group was significantly less likely to meet SUD criteria.

## Limitations

The small sample size was the major limitation of this study. A further shortcoming could rely on the fact that females (84%) and subject suffering from AN (52%) were disproportionally represented among this sample. Another drawback is the assessment using self-reported questionnaires. Finally, the cross-sectional character did not disclose about the direction of the relationship between mental illness and comorbid substance use.

## Conclusions

To our knowledge this is the first examination of prevalence, patterns, predictors and consequences of substance use among adolescent psychiatric inpatients in Austria. Our findings suggest that mentally ill minors, especially if suffering from externalizing or affective disorders or having a history of trauma, family dysfunction or substance using peers, are a population under high risk for elevated substance use and resulting psychosocial and health consequences. Early detection and prevention strategies focusing on these individuals are essential. Further studies using larger samples are now needed to further investigate this important issue.
